# Cell-Membrane-Coated Nanoparticles for Targeted Drug Delivery to the Brain for the Treatment of Neurological Diseases

**DOI:** 10.3390/pharmaceutics15020621

**Published:** 2023-02-13

**Authors:** Jianzhuang Li, Yanhao Wei, Chunlin Zhang, Rentang Bi, Yanmei Qiu, Yanan Li, Bo Hu

**Affiliations:** Department of Neurology, Union Hospital, Tongji Medical College, Huazhong University of Science and Technology, Wuhan 430022, China

**Keywords:** neurological diseases, cell membrane, nanoparticles, drug delivery, blood–brain barrier, targeting

## Abstract

Neurological diseases (NDs) are a significant cause of disability and death in the global population. However, effective treatments still need to be improved for most NDs. In recent years, cell-membrane-coated nanoparticles (CMCNPs) as drug-targeting delivery systems have become a research hotspot. Such a membrane-derived, nano drug-delivery system not only contributes to avoiding immune clearance but also endows nanoparticles (NPs) with various cellular and functional mimicries. This review article first provides an overview of the function and mechanism of single/hybrid cell-membrane-derived NPs. Then, we highlight the application and safety of CMCNPs in NDs. Finally, we discuss the challenges and opportunities in the field.

## 1. Introduction

NDs are a crucial cause of disability and death in the global population, placing a cumbersome burden on people. Over the past 25 years in particular, the burden of NDs has increased substantially due to an increasing population size and aging [1]. Moreover, environmental pollution from industrial production and global warming increase the risk of people developing NDs due to the probability that they are exposed to air pollutants and toxicants, in addition to the exacerbation of NDs by rising temperatures [2,3,4,5]. However, there is currently a remarkable lack of well-established clinical, therapeutic strategies for preventing or treating the progression of NDs due to the presence of the blood–brain barrier (BBB) [6]. The BBB is an essential feature of the central nervous system (CNS), limiting the entry of the vast majority of chemicals into the CNS, sequestering toxic chemicals away from the CNS, and guaranteeing the proper functioning of the CNS [7]. The BBB is the biggest obstacle to delivering drugs into the brain [8]. Therefore, there are two alternative directions. On one hand, increasing the permeability of the BBB to drugs by disrupting it does not lead to the accumulation of drugs specified in the brain [9,10]. Instead, it may increase the risk of harmful substances entering the CNS and causing irreversible damage. Although the BBB is damaged in various pathological, ND conditions, such as ischemic stroke (IS) [11], traumatic brain injury (TBI) [12], multiple sclerosis (MS) [13], glioblastoma multiforme (GBM) [14], Parkinson’s disease (PD) [15], and Alzheimer ‘s disease (AD) [16], it is also a significant challenge for the accumulation of drugs in the brain due to the unneglectable defects of current clinical CNS drugs, such as low solubility, a short half-life, and low permeability to the BBB [8]. On the other hand, the drug properties may be are altered to allow the drug to cross the BBB, which is the direction most researchers choose.

The emergence of NPs has identified some feasible solutions to this challenge. With their robust plasticity, NPs can increase drug accumulation in the CNS and are well-suited as carriers for the delivery of CNS drugs due to an artificial modification to increase solubility, stability in blood circulation, and the targeting properties of NPs [17,18]. However, NPs still have many defects. Repeated administration induces an immune response against NPs, resulting in the rapid clearance of NPs by the body’s immune system [19,20,21]. Moreover, the NPs themselves have a potential toxicity [22]. Some NPs have been demonstrated to cause oxidative stress and an inflammatory response after entering blood circulation [23,24]. For example, carbon-based NPs demonstrate toxic effects on healthy tissues in a long-term exposure state [25]. In addition, the preparation of nanomedicines is very dependent on artificial modification, resulting in a relatively complicated process of trial and a low preparation efficiency [21]. Therefore, NP-based targeted therapy has not yet exerted its full potency, and new directions are required to solve the problems posed by these defects. In targeting these defects, scientists have begun to design cell-membrane-coated nanoparticles (CMCNPs). CMCNPs typically adopt a core–shell design, covering a preformed NP core with a layer of cell membrane. After combining a cell membrane with novel nanoparticles, CMCNPs not only retain the advantages of NPs, but also increase cytocompatibility to protect the NPs from clearance by the body’s immune system. Prolonged drug circulation in the bloodstream significantly increases the drug’s BBB-crossing opportunity, enabling more drug accumulation in the brain [26]. In facing this biggest obstacle for delivering drugs into the CNS through the BBB, CMCNPs exploit the movement of cells in the body to achieve the drug-delivery purpose [27,28]. During an inflammatory response in the brain, chemokine levels increase in the brain lesions. For example, leukocyte CMCNPs exploit the chemotactic ability of leukocytes to target inflammation in the brain [29]. CMCNPs retain receptors for leukocyte membranes, adhere as tightly to brain endothelial cells (BECs) as leukocytes do, and migrate toward intracerebral lesions, driven via the chemokine–chemokine receptor pathway [29,30]. Moreover, CMCNPs further enhance a drug’s ability to target organs or tissues and improve therapeutic efficacy [31]. For example, the insertion of a ^D^WSW peptide into an RBC membrane surface by lipid insertion increases the targeting of the BBB by CMCNPs [32]. The insertion of NGR peptides into the RBC membrane surface strengthens the ability of drugs to target tumor tissues after crossing the BBB [32]. Many CMCNPs have shown strong efficiency in crossing the BBB and enhancing the power of drugs to accumulate, specifically in lesions [33,34,35,36,37]. Thus far, various types of cell membranes, such as the red cell membrane, white cell membrane, platelet membrane, cancer cell membrane, and others, have been used to research the fabrication of CMCNPs for treating NDs. In this review, as far as possible, we collect relevant studies from recent years regarding the application of CMCNPs in NDs and review the specific pathways of NP delivery. We believe that this review will help researchers to quickly understand recent advances regarding the use of CMCNPs in treating NDs, and shed light on their research.

## 2. Cell Membrane Types Currently Being Used to Coat Nanoparticles to Cross the BBB

The cell types currently used for the targeting of NDs by CMCNPs can be divided into two categories: one in which a single cell membrane is applied, and another in which two or even more species of the cell membrane are hybridized, discussed below in the classification.

### 2.1. Single Species of Cell Membrane

A single species of CMCNP is currently a relatively broad research class. There are many kinds of cells in the body’s interior, and each class of cells has cell membranes with different functions according to their unique physiological functions. Among them, red blood cells (RBCs) and white blood cells (WBCs) are the most thoroughly investigated cell types in the research of CMCNPs. In addition, there is research on platelets, stem cells, and abnormal cell types in the human body, such as cancer cells.

#### 2.1.1. Red-Cell-Membrane-Coated Nanoparticles (RCMCNPs)

RBCs have the following advantages for fabricating CMCNPs. Firstly, RBCs are the most abundant cellular components of human blood. They lack nuclei and mitochondria, so the RBC membranes are easy to extract [38]. Secondly, the brain is an organ with a relatively rich blood supply in the body, and RBCs have the ability to carry NPs across the BBB unobstructed. Thirdly, the normal physiological cycle of RBCs is between 100 and 120 days, which can guarantee that RCMCNPs have a longer circulation life in vivo [38]. In addition, cell-surface-specific functional proteins are retained when fabricating RCMCNPs, which avoids NPs being cleared out by the reticuloendothelial system (RES) as foreign materials. For example, it has been demonstrated that splenic macrophages rapidly clear RBCs lacking CD47 but do not clear RBCs that express CD47 by binding to the inhibitory receptor signal regulatory proteinα (SIRPα) [39]. Therefore, applying the RBC membranes, which usually express CD47, is an excellent idea for fabricating RCMCNPs.

However, RBCs lack targeting ability for NDs or brain lesions, which limits the use of RBCs in NDs. In response to this defect, researchers developed the idea of modifying the RBC membranes with ligands. As chemical modification can potentially damage specific functional proteins of RBC membranes and destroy the stability of the membrane’s integrity, researchers generally adopt the method of lipid or peptide insertion to increase the targeting ability of RCMCNPs [32,40,41,42].

**Figure 1:**
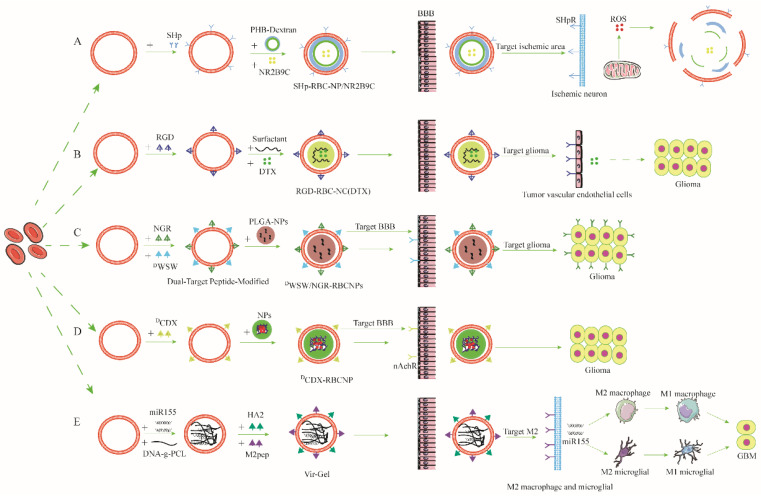
The preparation and characterization of red-cell-membrane-coated nanoparticles for ND drug delivery. (**A**) The preparation and characterization of SHp-RBC-NP/NR2B9C for ischemic stroke drug delivery; (**B**) the preparation and characterization of RGD-RBC-NC(DTX) for glioma drug delivery; (**C**) the preparation and characterization of ^D^WSW/NGR-RBCNPs for glioma drug delivery; (**D**) the preparation and characterization of ^D^CDX-RBCNP for glioma drug delivery; and (**E**) the preparation and characterization of Vir-Gel for glioblastoma drug delivery.

Figure 1 represents the composition of five related RCMCNPs [32,40,41,42,43]. Below, we illustrate the design thinking and delivery strategy of RCMCNPs using the research hosted by Wei Lv et al. [40]. Wei Lv et al. designed a type of RCMCNP, SHP-RBC-NP/NR2B9C, which targets the ischemic area. NR2B9C is a neuroprotective agent which prevents N-methyl-D-aspartate receptor (NMDAR)-mediated, ischemia-induced neurotoxicity without affecting the primary NMDAR activity [44,45]. However, it is a hydrophilic peptide with a considerable molecular weight, meaning it can hardly pass through the cell membrane and BBB [46]. Therefore, Wei Lv et al. adopted a strategy of coating drugs with RBC membranes to transport NR2B9C through the BBB. The experimental results showed that the efficiencies of the three free NR2B9C, NP/NR2B9C, and RBC-NP/NR2B9C groups to cross the BBB at two hours in vitro were 0.53%, 1.44%, and 1.71%, respectively [40]. This illustrates that cell membrane coating facilitates drug delivery across the BBB into the CNS. Next, to address the delivery issue of RCMCNPs, the researchers chose to insert the stroke homing peptide (SHP) into the RBC membrane to enable RCMCNPs to target the ischemic area. The SHP has been experimentally shown to selectively target sites of cerebral ischemia [47,48]. Furthermore, to control the release of drugs, they synthesized a reactive oxygen species (ROS) bio-responsive polymer, PHB dextran, to prepare the drug-carrying NPs. Due to the fact that IS causes a large amount of ROSs to be generated in the ischemic area of the brain, high levels of ROSs degrade PHB dextran, not only allowing the NPs to release NR2B9C but also alleviating ischemic damage by depleting the ROSs when NPs reach the ischemic area [40]. In this research, a relatively well-established drug delivery system was established, and the materials and ingredients applied were also very purposeful and fairly well-established.

#### 2.1.2. White-Cell-Membrane-Coated Nanoparticles (WCMCNPs)

WBCs, a relatively common cell subset in blood circulation, are more significant in volume than RBCs but belong to nucleated cells and are thus more difficult to extract than RBCs [49]. However, this does not affect the role of WBCs in the field of CMCNP research because the leukocyte has a targeting ability without any modification [50]. When NDs cause inflammation, WBCs, such as neutrophils, monocytes/macrophages (MMs), and lymphocytes, target the site of inflammation via the interaction between chemokines and chemokine ligands and participate in inflammation progression [51,52]. Among the cell species in which WBC membranes are utilized to fabricate WCMCNPs are mainly neutrophils and MMs (Table 1) [27,28,29,33,34,35,36,37,53,54,55,56,57,58,59].

Neutrophils comprise more than 50% of the total leukocyte population. When acute inflammation occurs, neutrophils tend to be the first responders to the site of inflammation [49,60]. For example, when IS onsets, neutrophils tend to appear in the ischemic area within an hour [60]. Because of the rapid response of neutrophils, they have enormous potential in the application of neutrophil membranes to fabricate targeted delivery systems of CMCNPs for the treatment of IS. Interestingly, researchers have exploited NPs to specifically target endogenous neutrophils, which are then carried by neutrophils into the ischemic area [34]. Of course, the premise is that NPs are not cleared by neutrophil digestion.

MMs are also an essential component of WBCs, playing an important role in infection, injury, inflammation, and tumors [50,61]. MMs are antigen-presenting cells that fundamentally avoid drug clearance by the immune system. Two phenotypes of macrophages, M1 and M2 [62], are particularly important to note. M1s are pro-inflammatory macrophages that can secrete various pro-inflammatory cytokines, contributing to the clearance of pathogens and damaged, unhealthy tissues [63]. The M2 type is an anti-inflammatory macrophage frequently found in tumor tissues. It is tightly associated with tumor growth and metastasis [63,64]. In addition, macrophages have a more vital ability to move within tissues. Based on these properties, MMs are often used to make drug delivery systems to treat neuroinflammation, brain gliomas, and brain metastases [27,36,37,56].

#### 2.1.3. Platelet-Cell-Membrane-Coated Nanoparticles (PCMCNPs)

Platelets, like RBCs, are a type of cell without a nucleus, so the extraction of platelet membranes is also easier. However, platelets are the smallest circulating blood cells in the bloodstream, and they are far less numerous in blood circulation than RBCs. They have a lifespan of only 8–9 days, leading to fewer applications in the fabrication of CMCNPs than other blood cells [65]. However, platelets have a natural advantage over RBCs. Platelets are essential in vascular injury, wound healing, inflammatory response, thrombosis, and hemostasis [66]. Therefore, it is advantageous to utilize platelet membranes over RBC membranes to coat NPs for the treatment of NDs with a collateral vascular injury or inflammatory response [67,68,69]. Moreover, besides the expression of CD47, there are some expression clusters on the surface of platelet membranes that inhibit the immune system. These include CD55, and CD59, which enhance the ability of immune evasion [70,71]. The lower immunogenicity of platelets compared to other nucleated cells, in addition to their immune evasion ability, suggests that the immune system would not clear them quickly after entering the blood circulation of PCMCNPs [72].

**Figure 2:**
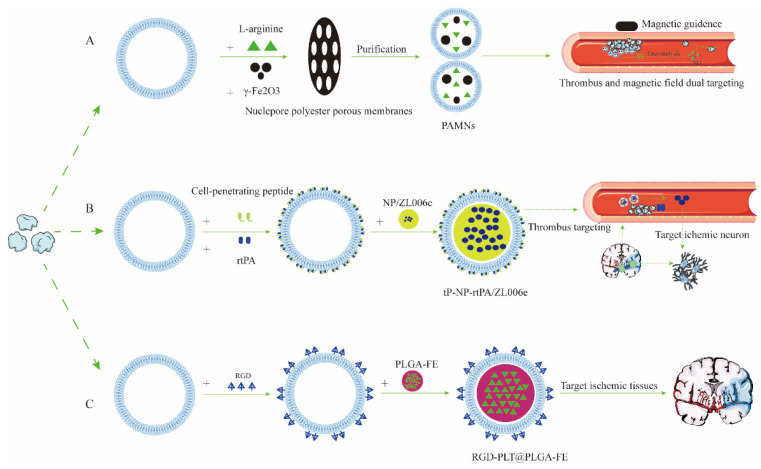
The preparation and characterization of platelet-cell-membrane coated nanoparticles for ND drug delivery. (**A**) The preparation and characterization of PAMNs for ischemic stroke drug delivery; (**B**) the preparation and characterization of tP-NP-rtPA/ZL006e for ischemic stroke drug delivery; and (**C**) the preparation and characterization of RGD-PLT@PLGA-FE for ischemic stroke drug delivery.

Based on the close interaction between platelets and strokes, there have been many studies to design stroke-targeted drug delivery systems (Figure 2) [67,69,73]. For example, Wang C. et al. recently designed a new type of PCMCNP: RGD-PLT@PLGA-FE [73]. Fat extract (FE) has been shown to be effective in treating IS, but its short half-life and inability to cross the BBB limit its use [74]. PCMCNPs improved the embarrassing status of this drug. Platelet membranes are inherently unique for IS and can actively target damaged or neovascular sites [75]. However, researchers also modified platelet membranes with RGD via lipid insertion to enhance the drug delivery system’s targeting ability. RGD not only specifically targets damaged and nascent blood vessels by interacting with vascular surface expressed α_Ⅴ_β_3_ integrin, but also increases the circulation time of drugs [76,77]. When the drug reaches the targeted site, the degradable, biomaterial-fabricated PLGA NPs sustainably release FE, achieving the effect of treating IS [78]. Although this is not a controlled drug-release method, this cannot negate the fact that PCMCNPs have excellent potential and advantages in developing a drug-targeted delivery system for IS.

#### 2.1.4. Cancer-Cell-Membrane-Coated Nanoparticles (CCMCNPs)

Cancer cells have properties many blood cells do not have. Firstly, cancer cells have the ability to proliferate indefinitely [79]. Therefore, the acquisition of cancer cell membranes does not necessarily require access to the body; cancer cells can also be acquired by culturing in vitro, which means that cancer cell membranes can be easily accessed [80,81,82]. Secondly, cancer cells have immune-evasion capabilities. CD47 is frequently overexpressed on the surface of cancer cells [83]. Thirdly, cancer cells have a homologous targeting ability [84]. Cancer cells mostly show homotypic aggregation, and homotypic cancer cells gather together [84]. Using cancer cell membranes to fabricate drug-targeting delivery systems makes cancer cell membranes carry NPs to target cancer tissues via Thomsen–Friedenreich antigen-galectin-3 interactions [85,86]. However, there are drawbacks associated with this property, which limits its application to cancer cell membranes. Currently, it is known that cancer-cell-membrane-coated nanoparticles are used on gliomas and metastases, as is detailed in Table 2 [80,81,82,85,86,87,88,89,90].

#### 2.1.5. Stem-Cell-Membrane-Coated Nanoparticles (SCMCNPs)

Thus far, the only research on the use of stem cell membranes to coat NPs was designed and carried out by Ma J et al. [91]. Apart from this research, we could not find other articles about using SCMCNPs for targeted drug delivery to the CNS.

The use of the stem cell membrane alone means simply applying the function of the cell membrane. The differentiation capacity of the stem cells cannot be applied. Therefore, there are many similarities with the utilization of leukocyte membrane coatings. This research chose glyburide as a therapeutic agent, which hitchhiked on the SCMCNPs. It has been demonstrated that glyburide can decrease infarct volume, edema, and hemorrhagic transformation and improve stroke outcomes. However, it lacks the ability to cross the BBB [92,93]. Therefore, researchers applied neural stem cells (NSCs) to fabricate CMCNPs for targeted drug delivery to the ischemic area. Moreover, to ensure that the CMCNPs could target the stroke area, they applied a viral transformation to over-express CXCR4 on the NSCs while culturing them. SDF-1 levels increased in the ischemic tissues after IS [94]. It is more efficient to use the interaction between SDF-1 and CXCR4 to drive CMCNPs to target the ischemic area [94,95]. While extracting the NSC membrane, the NSC membranes that overexpressed CXCR were isolated as the experimental group, while NSCs without CXCR overexpression were used as the control group. The results suggested that the accumulation of drugs in the ischemic area of the experimental group was more than twice that of the control group. In addition, this method of treating cells by viral transformation not only endowed the cell membrane with a targeting ability but also did not disrupt the integrity of the cell membrane, guaranteeing the prolonged circulation of NPs in the body more easily. Furthermore, to control the release of the drug, researchers chose an absorbable polymer PLGA to construct PLGA NPs, which cause piggyback drugs to constantly release over time [78,91].

#### 2.1.6. Neural-Cell-Membrane-Coated Nanoparticles (NCMCNPs)

Thus far, the only research on using neural cell membranes to coat NPs was designed and carried out by Zhang N, et al. [96]. Apart from this research, we could not find other articles about NCMCNP used in targeted drug delivery to the CNS. In the study, four kinds of neural cells, microglial, astrocytes, oligodendrocyte progenitor cells (OPCs), and cortical neurons, were applied to coat fluorescently trackable DPP-PCL NPs to explore an effective and targeted drug delivery system [96,97]. They successfully fabricated four kinds of NCMCNPs. When compared with uncoated DPP-PCL NPs, all three cell membrane coatings, except for the cortical neuron cell membrane, prevented microglial activation. In addition, microglia barely caused microglial activation, creating a favorable microenvironment for regenerating injured nerves [98]. Subsequently, they observed the uptake efficiency of uncoated and membrane-coated DPP-PCL NPs between different CNS cell types. Finally, they demonstrated that neural cells respond to NCMCNPs but lack specificity for the specific membrane-coated carriers. Additionally, these NCMCNPs were only taken up by astrocytes. In conclusion, for four kinds of NCMCNPs, the overall performance of the microglial coating was the best. Future research on NCMCNPs may focus on microglia-membrane-coated NPs for targeted CNS drug delivery.

#### 2.1.7. Vascular-Endothelial-Cell-Membrane-Coated Nanoparticles (VECMCNPs)

The BBB is dominated by endothelial cells and consists of a complex, multicellular, dynamic barrier [99]. Vascular endothelial cells (VECs), a constitutive component of the BBB, can serve as a raw material for the fabrication of CMCNPs [100]. In addition, some studies have shown that VECs also have a self-targeting ability [101,102]. Therefore, it might be possible to apply VEC membranes to fabricate CMCNPs targeted through the BBB to accumulate drugs in the brain.

Three studies have used vascular endothelial cells to fabricate CMCNPs for the targeted CNS delivery of drugs [103,104,105]. For example, DMSN-DHA@BMECM was designed to carry dihydroartemisinin (DHA) into the brain to protect against experimental cerebral malaria (CM) [105]. In the drug-targeting delivery system, vascular endothelial cell (VEC) membranes were responsible for carrying drugs across BBB and specifically targeted Plasmodium falciparum erythrocyte membrane protein 1 (PfEMP1), which is expressed on the surface of RBCs infected with Plasmodium falciparum [105,106]. Biodegradable, mesoporous silica nanoparticles (DMSN) were responsible for the drug release process [107]. The experiment was very ingenious in its application of the targeting ability of VEC membranes without modification of the cell membrane. In the future, researchers should focus on ingenious uses of cell membranes.

### 2.2. Hybrid-Cell-Membrane-Coated Nanoparticles (HCMCNPs)

**Figure 3:**
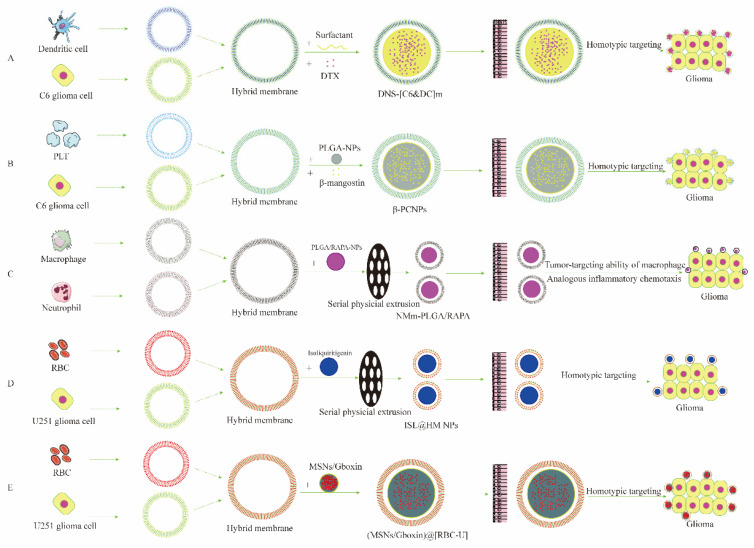
The preparation and characterization of hybrid-cell-membrane-coated nanoparticles for ND drug delivery. (**A**) The preparation and characterization of DNS-[C6&DC]m for glioma drug delivery; (**B**) the preparation and characterization of β-PCNPs for glioma drug delivery; (**C**) the preparation and characterization of NMm-PLGA/RAPA for glioma drug delivery; (**D**) the preparation and characterization of ISL@HM NPs for glioma drug delivery; and (**E**) the preparation and characterization of (MSNs/Gboxin)@[RBC-U] for glioma drug delivery.

In recent years, multifunctional hybrid cell membranes (HCMs) have begun to be used to fabricate HCMCNPs [108,109]. HCMs mostly employ two types of cell membranes to hybridize such that the HCMs possess unique biological functions. The types of HCMs for current applications in NDs are shown in Figure 3, and they include neutrophils–macrophages [110], dendritic cells–cancer cells [108], erythrocytes–cancer cells [111,112], and platelets–cancer cells [109]. HCMCNPs inherit the advantages of various cell membranes and make up for the deficiencies of different cell membranes. For example, the hybridization of platelet membranes, which have a robust immune evasion ability, with cancer cell membranes reduces the drug clearance by immune system and exploits the homologous targeting ability of cancer cell membranes [109]. It should be noted that the current drug delivery system targeting NDs, no matter which cells are hybridized with cancer cells, is applied to brain tumors, and there are no related reports regarding its application to other NDs [108,109,110,111,112]. Moreover, such designs must also investigate the effect of the ratio between different cell membranes on the drug-targeting delivery systems. It is also important to note whether the integrity of the original cell membranes is damaged during preparation, which may lead to the loss of function of the cell membrane.

## 3. Application of Cell Membrane Coated Nanoparticles in NDs

### 3.1. Ischemic Stroke

Ischemic stroke is a severe and lethal disease with a high prevalence and disability rate [113,114]. Although prompt clinical intervention through intravenous thrombolysis and endovascular thrombectomy may achieve the recanalization of brain blood vessels, an ischemic cascade, including oxidative stress, excitotoxicity, inflammation, and the immune response, would lead to irreversible brain damage [115,116,117,118]. Most neuroprotective agents targeting these biochemical events currently cannot adequately protect neurons from the injury due to poor BBB permeability [119,120]. The emergence of nanotechnology-based drug delivery platforms, especially CMCNPs, offers a promising approach to drug therapy for IS. Dong et al. reported a drug delivery platform consisting of neutrophil-membrane-derived nanovesicles loaded with RvD2 [35]. This nano-platform was inspired by the interaction between inflamed brain microvascular endothelium and neutrophils (the most abundant leukocytes in blood) during IS [35]. It explicitly targets the inflamed cerebrovascular system and enhances the delivery of RvD2. RvD2 induces nitric oxide production in endothelial cells for reduced leukocyte–endothelium interactions and stimulates neutrophil apoptosis, thereby accelerating the resolution of inflammation during ischemic stroke treatment [35,121,122]. Based a similar inspiration, a neutrophil-like, cell-membrane-wrapped mesoporous Prussian blue nanozyme (MPBzyme@NCM) was designed and developed in a 2021 study [33]. The coated, neutrophil-like cell membrane enhanced the delivery efficiency of nanomaterials to brain parenchyma and achieved a noninvasive, targeted nanozyme treatment for ischemic stroke [33]. After adhesion to inflamed brain vasculature, MPBzyme@NCM was phagocytized by microglia, thereby promoting microglia polarization toward M2, decreasing the recruitment of neutrophils and the apoptosis of neurons [33]. In a recent study, a PLT-membrane-coated nanocarrier loaded with l-arginine and γ-Fe2O3 magnetic NPs (PAMNs) was fabricated [67]. In addition to having good magnetic properties, the PAMNs are characteristic of the natural affinity between PLTs and the plaque thrombus in the damaged blood vessel. This study showed that the PAMNs could rapidly localize at ischemic brain sites with an efficient delivery of l-arginine. Subsequently, the production and release of NO at the lesion site increased, which promoted vasodilation and disrupted local PLT aggregation to delay the progression of thrombotic plaques [67]. In another study, platelet membranes were again used to coat the nanocarriers [73]. However, this PLT-membrane-cloaked nanocarrier delivered human FE to the infarct area of the stroke [73]. By targeting damaged and inflamed blood vessels, the nanocarriers loaded with FE (containing various angiogenic factors, such as VEGF, TGF-β, bFGF, and GDNF) rapidly converged on the lesion area of the ischemic brain, contributing to blood flow increase and neurobehavioral recovery [73]. CXCR4-overexpressing, cell-membrane-coated nanoparticles are another biomimetic drug delivery system commonly used in stroke treatment. This treatment depends on the interaction between the chemokine receptor CXCR4 and its ligand, SDF-1. For example, Ma et al. designed CXCR4-overexpressing, neural-stem-cell-membrane-coated nanoparticles to deliver glibenclamide (an anti-edematous agent) to ischemic areas [91]. Luo et al. fabricated biomimetic, smart nanoparticles comprising a CXCR4-overexpressing primary mouse thoracic aorta endothelial cell (PMTAEC) membrane envelope loaded with rapamycin (RAPA) [103]. These CXCR4-overexpressing CMCNPs had a high rate of target delivery efficacy into the ischemic brain tissues, representing a potentially promising approach for drug delivery [91,103].

### 3.2. Brain Tumors

GBM is the most frequent and aggressive malignant type of CNS tumor [123]. Despite surgical resection and adjuvant therapies, the median survival rate for GBM patients is only 14.6 months due to the specific tumor location and the formidable obstacle of the BBB and the blood–brain tumor barrier (BBTB) [37,124,125]. Thus, it is desperately urgent to explore novel drug delivery systems for treating GBM. Various cell membranes have been shown to encapsulate nanoparticles to facilitate the delivery of anti-glioblastoma drugs, including RBC membranes, platelet cell membranes, macrophage cell membranes, and cancer cell membranes. Among the diverse cell membrane types, tumor cell membranes stand out because of their homotypic targeting property. The property endows the nanoparticles wrapped in tumor cell membranes with a tumor-targeting ability [126,127,128,129]. Fan et al. reported a kind of glioma-C6-cancer-cell-membrane-coated paclitaxel nanosuspensions ((PTX)NS) which was able to traverse across the BBB effectively and target tumor tissues selectively [87]. In a recent study, an engineered macrophage-membrane-coated nano-platform was successfully developed, and it showed enhanced programmed cell death-1 (PD-1) expression [37]. This nano-platform could efficiently cross the BBB into the brain in response to the immune tumor microenvironment (iTME) recruitment and ultimately accumulated at the tumor site [37]. Considering the low immunogenicity of erythrocyte cell membranes, Cui et al. developed erythrocyte-membrane-coated nanoparticles with modified DWSW and NGR peptide ligands [32]. This dual-targeting nanocarrier was capable of traveling across the BBB and the BBTB efficiently and targeting glioma cells [32]. Integrating different cell membranes into hybrid membranes provides a promising approach to develop multifunctional cell-membrane-based nanoplatforms [130]. Platelet–tumor and erythrocyte–tumor cell hybrid-membrane-coated nanoparticles have been designed and developed for anti-glioblastoma drug delivery [109,111,112]. These biomimetic hybrid-membrane-based nanoplatforms demonstrate excellent tumor targeting and immune escape capabilities to enhance the therapeutic outcome [109,111,112]. For example, Shi et al. designed and fabricated a hybrid-membrane-camouflaged nano-platform consisting of erythrocytes and tumor cells [111]. Such hybridized, membrane-derived nanocarriers significantly enhanced drug solubility, targeting, and antitumor effect [111].

### 3.3. Other NDs

AD is a progressive and deadly neurodegenerative disease [131]. Numerous research on AD therapy currently focuses on the β-amyloid protein (Aβ). Mounting evidence demonstrates that mitochondrial dysfunction is an early, critical pathologic event in the development of AD [132,133,134]. At the same time, the oxidative stress of neuronal mitochondria is associated with the generation and aggregation of Aβ [135,136]. Thus, neuronal mitochondria dysfunction may be a novel therapeutic target for AD. Han et al. fabricated macrophage-membrane-wrapped NPs loaded with rabies virus glycoprotein (RVG29) and triphenylphosphine cation (TPP) molecules for delivering functional antioxidant-genistein (GS) to neuronal mitochondria [57]. The biological characteristics of the macrophage membranes provided good biocompatibility and immune evasion ability in the circulation of the nano-system [57]. The membrane-loaded RVG29 and TPP collaboratively enabled the GS-encapsulated nano-system to cross the BBB, target neurons, and subsequently enter the neuronal mitochondria, ultimately leading to effective relief of AD symptoms [57].

Malaria is among the top three most deadly human infectious diseases [137,138]. Notably, 90% of deaths are attributed to CM, a severe CNS complication of malaria [139]. A recent study described a strategy for the therapy of experimental cerebral malaria by combining a biodegradable NP loaded with the antimalarial drug DHA using a brain microvascular endothelial cell (BMEC)-membrane [105]. The BMECM coating of the NPs enhanced the adhesion and targeting of the nanoparticle drug to the infected RBCs (iRBCs) due to the interaction between natural cell membranes and the pathogen-infected host cells [105]. Thus, this strategy, based on cell-membrane-derived NPs, improved therapeutic outcomes in an experimental cerebral malaria model.

## 4. Safety and Outcomes after CMCNPs Enter the Circulation

### 4.1. The Safety of CMCNPs

In the above sections, we reviewed various cell-membrane-coated NPs and their applications in the treatment of different NDs. Next, we will discuss the in vitro cytotoxicity assay and in vivo biodistribution, as well as the tissue damage caused by CMCNPs after they enter the blood circulation.

After coating NPs with the cell membrane, the time length spent by the drug in circulation increases. This may cause toxic damage to healthy tissues [37]. Many studies have shown that the most vulnerable sites for the accumulation of CMCNPs after their entry into blood circulation are the lesions, liver, lung, and spleen; however, CMCNPs do not cause damage to these tissues [28,33,34,35,36,37,57,58,59,80,87,90]. In addition, in most reports about CMCNPs, the toxicity testing of CMCNPs in vitro and vivo showed that the cytotoxicity and systemic toxicity of CMCNPs were negligible [29,34,36,54,81,90]. Using MPM@P NGs to treat C6 cells and bEnd.3 cells, respectively, the results showed that the cytotoxic ability of the MPM@P NGs for C6 cells was much higher than that of the bEnd.3 cells [36], illustrating that MPM@P NGs were less toxic to normal cells. This could be attributed to the ability of MPM@P NGs to target lesions. Therefore, cisplatin accumulation was found in liver tissues after ten days of treatment using NGs when not coated with the cell membrane. Meanwhile, liver tissues presented acute necrosis and inflammatory cell infiltration [36]. This also illustrates that the release of nanoparticles might cause pathological damage to normal tissues after the off-target occurrence of effects of CMCNPs. However, a different scenario emerged in another study. Chen et al. injected five groups of mice with saline, free DHA, DHA-NLC, DHA-NLC, or DHA-NGR/CCNLC for 15 days. The results indicated that there was no apparent tissue damage in the organs of mice in each group. Additionally, all the indexes of the blood routine and blood biochemistry of the mice in each group were within normal limits, illustrating the excellent biocompatibility of the CMCNP and that even if off-target effects occurred after the CMCNP entered circulation, they would not cause apparent tissue damage to the body [81]. The difference in the two scenarios was likely because this CMCNP-loaded drug, DHA, and the NP’s material were comparatively less toxic [140,141]. Therefore, in future studies on CMCNPs, the NP material that is hitchhiked by the drug should be selected from materials with high efficiency and low toxicity, as the toxicity of the drug itself is sometimes unavoidable when treating certain diseases.

### 4.2. The Clearance after CMCNPs Enter the Blood Circulation: Three Scenarios

#### 4.2.1. Normal Condition

Normally, CMCNPs have two targeting functions: targeting the BBB and targeting the lesion area [142]. Ideally, after CMCNPs enter the blood circulation, most CMCNPs cross the BBB, target the lesion area, are internalized by the cells of interest, and then release the loaded drug so that the lesion area accumulates the concentration needed for treatment. After CMCNPs unload the drug and exert their effect, a final consideration is how to clear the CMCNPs from the CNS.

The cellular uptake of nanomaterials has been found in neurons, dendritic cells, and glial cells. Therefore, intracellular degradation, particularly in lysosomes, may be a mechanism for clearing nanomaterials by the central nervous system [143,144,145]. Microglia, as immune cells of the CNS, may be the essential cell type mediating the intracellular degradation of NPs in the CNS [146,147]. Metabolic enzymes in the brain parenchyma also participate in the extracellular degradation of NPs [148]. Moreover, the application scenario of CMCNPs mainly occurs at the onset of NDs, when neuroinflammation mostly occurs within the lesion, massive microglial activation occurs, and the recruitment of peripheral immune cells occurs, which also leads to the intracellular clearance of NPs [51,52]. In addition, NPs may be cleared from the CNS to the blood or cervical lymphatics via CSF circulation through the paravascular pathway and functional lymphatic vessels; this might be mainly used for the clearance of non-degradable NPs [149,150,151,152]. The clearance mechanisms of CMCNPs have been scarcely addressed in the current studies on CMCNPs. Therefore, future research may focus on the clearance mechanisms of CMCNPs.

#### 4.2.2. Off-Target Effects of CMCNPs before Crossing the BBB

If CMCNPs do not enter the CNS after they enter the blood circulation, NPs expose the disadvantages discussed above and are rapidly cleared by the collective immune system if the cell membrane coating ruptures. For example, ^D^WSW-CCM-(PTX)NS, in addition to accumulating most in the brain lesions, also had a higher fluorescence concentration in the liver and spleen [87]. This phenomenon may be related to the role of hepatic Kupffer cells versus the spleen, in which macrophages capture NPs released in the blood circulation and traffic them to the liver and spleen for elimination [82,87,127].

#### 4.2.3. Off-Target Effects of CMCNPs after Crossing the BBB

The last scenario occurs when CMCNPs cross the BBB after entering the blood circulation and are moved off-target into healthy brain tissues instead of being targeted for movement to the brain lesions. In this scenario, CMCNPs unload the loaded drug in normal tissues. If the drug is not cleared on time, the potential exists for irreversible damage to healthy brain tissue. If damage occurs, leading to local inflammation, the pathways for clearance are similar to those in the first case. If the drug is cleared in time without causing tissue damage, there is no clearance of NPs by peripheral immune cells, and the rest of the process is similar to the first scenario.

Therefore, to prevent the emergence of off-target situations, improving the quickly cleared nuclear content properties should be considered when designing CMCNPs. Meanwhile, the ability of CMCNPs to target the BBB and brain lesions will also be enhanced by targeted modifications, which could be helpful in reducing the risk of developing pathological, off-target damage.

### 4.3. Potential Adverse Effects of CMCNPs on the Body

#### 4.3.1. Potential Adverse Effects of Cell Membrane Coatings

Various types of cell membranes, such as red cell membranes, white cell membranes, platelet membranes, cancer cell membranes, and others, have been used to research the fabrication of CMCNPs for treating NDs [33,43,59,73,90]. Although the removal of the nuclear fraction of the cell uses only the cell membrane, the physicochemical properties of the cell membrane are primarily preserved and have the potential to have some effect on the body’s immune system. Zhang et al. discovered that coating NPs with CNS cell membranes (including microglia, astrocytes, and oligodendrocyte progenitor cells) did not cause an apparent specific uptake of microglia but prevented microglial activation when compared to the uncoated NPs [96]. Interestingly, the most evident inhibition of microglia activation by microglial-membrane-coated NPs was observed [96]. Therefore, CNS-cell-membrane-coated NPs may reduce the immune response caused by microglia as well as unwanted side effects. In a study designed by Fan Y et al., treatment with cell-membrane-coated HCPT-NS/CCM demonstrated more stable WBC levels than compared to treatment without cell-membrane-coated HCPT-NS, demonstrating that an extra layer of cell membrane coating did not affect the body’s immune system [80]. However, there is now no direct evidence that cell membrane coatings have no effect on the immune system. Therefore, future research may focus on whether cell membrane coatings have the potential effects on the body’s immune system.

#### 4.3.2. Potential Adverse Effects of Long-Term Application of CMCNPs

Many CMCNPs are currently dose-dependent, although many studies have shown no cytotoxicity and systemic toxicity when treated with drugs at experimental doses. However, if CMCNPs are not cleared on time and CMCNPs in vivo exceed the experimental dose, they have the potential to cause pathological damage to the body, which is easily the case with the long-term application of drugs. For example, a study designed by Shen et al. showed that a gradual increase in the concentration of NV-CUR led to a gradual decrease in the neuroprotective effect [58]. If the body does not clear NV-CUR, it manifests as neurotoxicity at higher concentrations, accumulating within the CNS. However, the short duration of the study made it difficult to assess the potential adverse effects of the long-term application of CMCNPs. Therefore, future studies need to focus on whether the long-term application of CMCNPs can cause possible adverse effects on the body.

## 5. Summary and Perspective

Advances in nanomedicine show significant advantages in both efficacy and safety and offer opportunities for the targeted delivery of various therapeutic agents. However, there are still many research areas for such CMCNPs to be confirmed in. In most experiments, the release studies on drugs from membrane-coated NPs can only be performed through in vitro experiments, and it is not known how drug release occurs when CMCNPs reach the targeting site. Artificially controlling the release process of drugs has not been performed when targeting ND administration; this is a direction that could be studied. Additionally, none of applications of CMCNPs in the currently conducted animal experiments have had a very long duration. Although no apparent safety concerns have been observed with short-term use, the safety of the long-term application of such drug-targeted delivery systems cannot be predicted. Moreover, there have been no clinical studies of such nano-delivery systems. Future research should give full play to the advantages of multi-disciplinary research, improve the preparation and safety assessments of membrane-coated NPs, and carry out clinical trials as soon as possible.

## Figures and Tables

**Table 1:** Summary of white-cell-membrane-coated nanoparticles for ND drug delivery.

Drug Targeted Delivery System	Cell Membrane Source	Targeted Modification	Materials of NPs	Drug-Loading	Mechanism of Crossing the BBB	NDs Model	Reference
cl PGP-PEG-DGL/CAT-Aco system	Endogenous neutrophils	PGP/CXCR2	ACO NPs	Catalase (CAT)	Neutrophil-mediated transport through the BBB	MCAO	[34]
Neutrophil membrane-derived nanovesicles	Neutrophils	No	-	RvD2	Integrin β2 and PSGL-1 of neutrophils binding to inflamed endothelium	I/R	[35]
MPBzyme@NCM	Neutrophils	No	PVP and Fe[(CN_6_)]^3-^	MPBzyme	Integrin β2 and Mac-1 of neutrophils targeting to ICAM-1 of inflamed brain microvascular endothelial cells	IS	[33]
Monocytes loaded with liposomes	Monocytes	No	Liposomes	Serotonin	Monocyte-mediated transport through the BBB	No	[53]
IDV-NP-BMM	Bone marrow macrophages	No	IDV-NP suspensions	Indinavir (IDV)	Macrophage-mediated transport through the BBB	HIV-1 encephalitis	[54]
BMM-PEI-PEG/cat	Bone marrow macrophages	No	PEI-PEG	CAT	BMM adhesion to cerebral vascular endothelial cells	PD	[55]
BMM loaded with nanozyme	Bone marrow macrophages	No	PEI-PEG	CAT	BMM adhesion to cerebral vascular endothelial cells via integrin α4	PD	[29]
MMs loaded with therapeutic NPs	MMs	No	Gold nano-shells and silica particles	No	MMs-mediated transport through the BBB	Brain metastases of breast cancer	[56]
Macrophages loaded with nanozyme	Macrophages	No	PEI-PEG	Catalase	Macrophage-mediated transport through the BBB	Neuroinflammation/PD	[27]
Nanoparticles loaded NPs	Monocytes	No	Magnetite- laden NPs	No	Monocyte-mediated transport through the BBB	Epileptic	[28]
RVG/TPP-MASLNs	Macrophages	RVG29 and TPP	Solid lipid nanoparticles (SLNs)	Genistein (GS)	RVG29 peptide for crossing the BBB	AD	[57]
NVs-CUR	Macrophages	No	No	Curcumin (CUR)	Not mentioned	PD	[58]
McM/RNPs	Monocytes	No	PLGA-NPs	Rapamycin (RAPA)	Integrin α4 and integrin β1 of monocytes targeting to inflammatory endothelial cells	MCAO	[59]
MPM@P-NGS	Macrophages	No	MnO_2@PVCL NGS	Cisplatin	Cell-carrier-mediated BBB traversing based on endogenous immunocytes	Orthotopic glioma	[36]
PD-1-MM@PLGA/RAPA	Macrophages	PD-1	PLGA-NPs	Rapamycin	Macrophage-mediated transport through the BBB	GBM	[37]

**Table 2:** Summary of cancer-cell-membrane-coated nanoparticles for ND drug delivery.

Drug Targeted Delivery System	Cancer Cell Membrane	Targeted Modification	Materials of NPs	Drug-Loading	Cancer Model	Reference
CCNP	MDA-MB-831cells	No	mPEG-PLGA-NPs	Doxorubicin (DOX)	Brain and metastatic breast cancer	[82]
CM-NCubes	U-251 MG cells	No	Fe_3_O_4_/MnO_2_- NCubes	Sorafenib and AMF	GBM	[88]
Dox-CM-BNNTs	U87 MG cells	No	BNNTs	DOX	GBM	[89]
DHA-NGR/CCNLC	C6 glioma cells	NGR	NLCs	DHA	Glioma	[81]
WSW-CCM-(PTX)NS	C6 glioma cells	DWSW	PVP K30 and SDC nanosuspension	Paclitaxel (PTX)	Glioma	[87]
HCPT-NS/CCM	C6 glioma cells	No	Nanosuspension	10-hydroxycamptothecin (HCPT)	Glioma	[80]
MnO_2_-DOX-C6	C6 glioma cells	No	MnO_2_	Doxorubicin (DOX)	Glioma	[85]
HDX@YSN@CCM@cRGD	U87 MG cells	cRGD	YSNs	Hydroxychloroquine (HDX)	Glioblastoma	[86]
CC-LnNPs	U87 MG cells	No	LnNPs	No	Glioma	[90]

## Data Availability

Not applicable.

## Abbreviations

neurological diseases	NDs
cell membrane-coated nanoparticles	CMCNPs
nanoparticles	NPs
blood–brain barrier	BBB
central nervous system	CNS
ischemic stroke	IS
traumatic brain injury	TBI
multiple sclerosis	MS
glioblastoma multiforme	GBM
Parkinson’s disease	PD
Alzheimer’s disease	AD
brain endothelial cells	BECs
red blood cells	RBCs
white blood cells	WBCs
red cell membrane-coated nanoparticles	RCMCNPs
N-methyl-D-aspartate receptor	NMDAR
reticuloendothelial system	RES
signal regulatory proteinα	SIRPα
reactive oxygen species	ROS
stroke homing peptide	SHP
white cell membrane-coated nanoparticles	WCMCNPs
monocytes/macrophages	MMs
fat extract	FE
catalase	CAT
resolvin D2	RvD2
ischemia/reperfusion	I/R
genistein	GS
curcumin	CUR
indinavir	IDV
rapamycin	RAPA
platelet cell membrane-coated nanoparticles	PCMCNPs
cancer cell membrane-coated nanoparticles	CCMCNPs
doxorubicin	DOX
hydroxychloroquine	HDX
10-hydroxycamptothecin	HCPT
paclitaxel	PTX
stem cell membrane-coated nanoparticles	SCMCNPs
neural stem cells	NSCs
oligodendrocyte progenitor cells	OPCs
neural cell membrane-coated nanoparticles	NCMCNPs
vascular endothelial cells	VECs
cerebral malaria	CM
hybrid cell membrane-coated nanoparticles	HCMCNPs
primary mouse thoracic aorta endothelial cell	PMTAEC
blood-brain tumor barrier	BBTB
immune tumor microenvironment	iTME
β-amyloid protein	Aβ
rabies virus glycoprotein	RVG29
triphenylphosphine cation	TPP
infected RBCs	iRBCs
dihydroartemisinin	DHA
